# Mapping the Genetic Regions Responsible for Key Phenology-Related Traits in the European Hazelnut

**DOI:** 10.3389/fpls.2021.749394

**Published:** 2021-12-23

**Authors:** Nadia Valentini, Ezio Portis, Roberto Botta, Alberto Acquadro, Vera Pavese, Emile Cavalet Giorsa, Daniela Torello Marinoni

**Affiliations:** Dipartimento di Scienze Agrarie, Forestali e Alimentari (DISAFA), University of Turin, Turin, Italy

**Keywords:** quantitative trait loci, flowering time, dichogamy, nut maturity time, *Corylus avellana*

## Abstract

An increasing interest in the cultivation of (European) hazelnut (*Corylus avellana*) is driving a demand to breed cultivars adapted to non-conventional environments, particularly in the context of incipient climate change. Given that plant phenology is so strongly determined by genotype, a rational approach to support these breeding efforts will be to identify quantitative trait loci (QTLs) and the genes underlying the basis for adaptation. The present study was designed to map QTLs for phenology-related traits, such as the timing of both male and female flowering, dichogamy, and the period required for nuts to reach maturity. The analysis took advantage of an existing linkage map developed from a population of F_1_ progeny bred from the cross “Tonda Gentile delle Langhe” × “Merveille de Bollwiller,” consisting in 11 LG. A total of 42 QTL-harboring regions were identified. Overall, 71 QTLs were detected, 49 on the TGdL map and 22 on the MB map; among these, 21 were classified as major; 13 were detected in at least two of the seasons (stable-major QTL). In detail, 20 QTLs were identified as contributing to the time of male flowering, 15 to time of female flowering, 25 to dichogamy, and 11 to time of nut maturity. LG02 was found to harbor 16 QTLs, while 15 QTLs mapped to LG10 and 14 to LG03. Many of the QTLs were clustered with one another. The major cluster was located on TGdL_02 and consisted of mainly major QTLs governing all the analyzed traits. A search of the key genomic regions revealed 22 candidate genes underlying the set of traits being investigated. Many of them have been described in the literature as involved in processes related to flowering, control of dormancy, budburst, the switch from vegetative to reproductive growth, or the morphogenesis of flowers and seeds.

## Introduction

The European hazelnut (*Corylus avellana* L., *2n* = *2x* = *22*) is a high-value cash crop, with the two largest producers being Turkey and Italy, and growing industry in several other countries worldwide (Food and Agriculture Organization Corporate Statistical Database [FAOSTAT], 2021). The increased interest in this crop is fueling a demand for cultivars adapted to non-conventional environments, particularly in the context of incipient climate change. The phenological traits are considered key to adaptation (Ghelardini et al., 2014) and include the time of male and female flowering, budburst, and nut maturity.

The flowering behavior of hazelnut is somewhat unusual, as it occurs during the winter. In the northern hemisphere, pollen is shed and the pistils are receptive between mid-December and mid-March, dependent on genotype, year, and location (Mehlenbacher, 1991). Moreover, hazelnut plants are monoecious, wind-pollinated, and most cultivars are dichogamous, that is the male and female reproductive organs mature at different times. Because the species is self-incompatible and even cultivars are cross-incompatible in some parental combinations (Mehlenbacher and Thompson, 1988), it is necessary to include a pollinizer genotype in orchards, since insufficient pollination causes yield reduction. Flowering time in hazelnut, as is similarly the case for most plant species, is both under strong genetic control, and also strongly influenced by the environmental factors (Jung and Müller, 2009) and is a major factor to be considered in climatic adaptation both to warm and frosty areas (Ntladi et al., 2018). The time of budburst is another important limiting factor of the environment in which cultivars can be successfully grown. In pear (*Pyrus communis*), for example, the failure to satisfy the plant’s chilling requirement compromises vegetative budburst (Gabay et al., 2018).

In hazelnut, budburst dates widely differ in hazelnut cultivars, but in northern Italy, leaf emergence typically happens between mid-March and early April. Otherwise, nuts of the major hazelnut cultivar mature over a period from August to October. Early maturing cultivars are favored by growers, both because harvesting during the dry, warm weather typical of the late summer is easier than in autumn, and the nuts harvested under dry conditions are better able to maintain their high nutritional quality.

Conventional breeding in tree crops is a slow process, but it can be accelerated by the application of marker-assisted selection (MAS). The first genetic linkage map of hazelnut appeared 15 years ago (Mehlenbacher et al., 2006), but since this time others have been added (Gürcan and Mehlenbacher, 2010a,b; Gürcan et al., 2010; Beltramo et al., 2016; Bhattarai and Mehlenbacher, 2017; Colburn et al., 2017; Rowley et al., 2018; Torello Marinoni et al., 2018). The maps have been used to identify several major genes conferring resistance to Eastern Filbert Blight and self-incompatibility (Rowley et al., 2018), as well as to show that traits such as vigor, sucker habit, and timing of budburst are all under polygenic control (Beltramo et al., 2016; Torello Marinoni et al., 2018). Ozturk et al. (2017) exploited a genome-wide association (GWA) mapping approach to identify simple sequence repeat (SSR) markers associated with nut and kernel traits. Some hazelnut genomic resources are also available in the public domain, including its transcriptome (Rowley et al., 2012), a *de novo* assembled genome of the cultivar “Jefferson” and some resequencing data (Rowley et al., 2018)^1^. Very recently, a fully assembled and annotated genome sequence of cultivar “Tombul” was published (Lucas et al., 2021)^2^ as well as that of the cultivar “Tonda Gentile delle Langhe” (TGdL) (Pavese et al., 2021)^3^.

The present study was designed to determine the genomic regions associated with flowering, dichogamy, and nut maturity. The approach chosen was to apply quantitative trait locus (QTL) mapping to a set of F_1_ progeny bred from the cross TGdL × “Merveille de Bollwiller” (MB), which has previously been used to identify QTLs associated to the timing of budburst (Beltramo et al., 2016; Torello Marinoni et al., 2018). Based on available annotated genome sequences, these genomic regions were exploited to identify potential candidate genes underlying the phenology of hazelnut.

## Materials and Methods

### Plant Materials

A total of 275 seedlings of the F_1_ progeny bred from the cross TGdL × MB, described by Beltramo et al. (2016) and Torello Marinoni et al. (2018), and three individuals obtained from rooted suckers of each of the two parents, were planted at the campus of the University of Torino (Department of Agricultural, Forest and Food Sciences; 45°07′N; 7°58′E; 293 m a.s.l.) in 2009.

The mapping population was a set of 213 individuals, planted in the core of the field. The plants, spaced 4 × 4 m, and trained in an open vase system, were irrigated between mid-June and mid-September using an integral PC drip line (UniRam 20010 AS, Netafim). Local meteorological data (temperature, relative humidity, rainfall) were recorded by Regione Piemonte - Rete Agrometeorologica Regionale using an automatic weather station comprising a set of sensors installed 2 m above the ground, following World Meteorological Organization guidelines.

### Assessment of Phenology

Across four seasons (2012/13 to 2015/16), records were taken every 5–7 days from the end of December until mid-March with respect to the dates at which 10% of the catkins had released pollen (time of male flowering, *tmf*) and at which 10% of the female flowers were receptive (time of female flowering, *tff*), following Germain and Sarraquigne (2004). The data were grouped into nine classes according to International Union for the Protection of New Varieties of Plants [UPOV] (1979), from very early (1) to very late (9). The degree of dichogamy (*dc*) was scored on a scale of 1 (very protandrous) to 9 (very protogynous), following International Plant Genetic Resources Institute [IPGRI] et al. (2008). Time of nut maturity (*tnm*) was assessed across three seasons (2014–2016), with measurements being taken every 5–7 days from the end of July until the beginning of October, defined by the date at which 10% of the nuts had dropped from the tree. The data were converted into nine classes, from very early (1) to very late (9), following UPOV guidelines.

Population means, standard deviations, normality (kurtosis and skewness), and trait correlations were calculated using the IBM SPSS Statistics v25.0 package^4^. Normality, kurtosis, and skewness were tested using the Shapiro Wilks test (α = 0.05). Correlations between the traits, including the time of leaf budburst (*tlb*), as given by Torello Marinoni et al. (2018), were calculated on the basis of the Spearman coefficient. Segregation was considered as transgressive where the performance of at least one F_1_ individual either exceeded that of the higher scoring parent or fell short of that of the lower scoring parent by at least two standard deviations.

### Quantitative Trait Loci Detection

The QTL analysis was performed using MapQTL v5 software (van Ooijen, 2004), based on the two parental maps (TGdL and MB) developed by Torello Marinoni et al. (2018). The location of putative QTL was based initially on the simple interval mapping procedure (Lander and Botstein, 1989), then confirmed using the multiple QTL mapping procedure (Jansen and Stam, 1994). A mapping step size of 1 cM was used in both analyses. For the multiple QTL mapping, a backward elimination procedure was used to select appropriate co-factors (e.g., significantly associated with each trait at *p* < 0.02). Genome-wide logarithm of odds (LOD) thresholds (*P* < 0.05) were determined empirically for each trait, using the PERMUTATION test provided within MapQTL with 1,000 iterations (Churchill and Doerge, 1994). Only QTLs associated with an LOD higher than the genome-wide threshold were considered, and 1-LOD support intervals were determined for each LOD peak (van Ooijen, 1992). The proportion of the overall phenotypic variance (PV) associated with each QTL was estimated from the multiple QTL mapping model. QTL positions were drawn using MapChart (Voorrips, 2002). Each QTL was designated by its trait name (*tmf*, *tff*, *dc*, *tnm*, and *tlb*), followed by the relevant linkage group (LG) and the relevant season: thus, for example, *tmf_TGdL_02_13* indicates a QTL underlying *tmf* mapping to LG02 on the TGdL map, as identified from data collected in the 2012/13 season.

### Candidate Gene Detection

Markers for map development and QTL analyses were initially identified using the “Jefferson” genome (Torello Marinoni et al., 2018). Recently, the genome of the cultivar TGdL, containing a wider number of annotated genes, was publicly made available (Pavese et al., 2021). Thus, candidate genes were retrieved by mapping the “Jefferson” scaffolds containing QTLs on the TGdL genome (Pavese et al., 2021) using a BLAST search. The structural/functional annotation of the genes in the QTL was carried out using the gff annotation file provided by Pavese et al. (2021) (see text footnote 3). Genes in the QTL intervals were discussed when showing a clear function/annotation related to flowering-like processes, as inferred from literature.

## Results

### Phenotypic Data of Parental Cultivars

The set of phenological data for both the parents and the mapping population (Table 1) showed a significant degree of season-to-season variation (*P* < 0.05) between TGdL and MB for *tmf*, *tff*, and *tnm*, but not for *dc*. For TGdL, *tmf* was either “very early” (class 1) or “very early to early” (class 2), while *tff* ranged from “very early” (class 1) to “early” (class 3); for MB, *tmf* and *tff* were scored as, respectively, “medium-late” (class 6) to “late” (class 7). MB plants were consistently scored as “homogamous” (*dc*, class 5), while TGdL plants were “slightly protandrous” (class 4) in three of the 4 years, and “slightly protogynous” (class 6) in 2013. With respect to *tnm*, TGdL was scored as “very early to early” (class 2) in 2 of the 3 years and “early” (class 3) in 1 year, while MB was scored as either “medium” or “medium-late” (classes 5 to 6).

**TABLE 1:** Variation for time of male flowering (*tmf)*, time of female flowering (*tff)*, dichogamy (*dc*), and time of nut maturity *(tnm)*.

Trait	Year	Parents (Mean and SD)						Progeny (F1 population)							
		TGdL		MB		Wilcoxon test	Mid-parent value	Mean	*SD*	Range	*SE*	Skewness	*SE*	Kurtosis	*SE*
*Tmf*	2013	2.00	0.00	6.67	0.58	Yes *P* < 0.05	4.33	4.04	1.40	1–8	0.11	0.416	0.187	−0.144	0.373
	2014	2.00	1.00	6.33	0.58	Yes *P* < 0.05	4.17	4.28	1.19	1–8	0.08	0.070	0.174	0.995	0.346
	2015	1.00	0.00	6.67	0.58	Yes *P* < 0.05	3.83	4.24	1.82	1–9	0.13	0.548	0.168	−0.612	0.335
	2016	2.00	0.00	7.00	0.00	Yes *P* < 0.05	4.50	4.93	1.22	1–8	0.08	−0.368	0.167	0.696	0.333
tff	2013	1.33	0.58	6.33	0.58	Yes *P* < 0.05	3.83	4.70	1.81	1–8	0.13	−0.066	0.167	−1.237	0.333
	2014	3.00	0.00	6.67	0.58	Yes *P* < 0.05	4.83	5.10	1.37	2–7	0.09	0.399	0.167	−1.256	0.332
	2015	2.00	0.00	6.67	0.58	Yes *P* <0.05	4.33	4.82	2.11	2—9	0.14	−0.356	0.167	− 0.429	0.333
	2016	2.33	0.58	7.00	0.00	Yes *P* < 0.05	4.67	5.45	1.45	2—9	0.10	−0.020	0.189	−0.223	0.376
*dc*	2013	5.67	0.58	5.33	0.58	ns	5.50	4.51	1.64	1–9	0.13	−0.047	0.168	−0.248	0.335
	2014	4.00	1.00	4.67	0.58	ns	4.33	4.26	1.34	1–9	0.10	0.136	0.167	−0.297	0.333
	2015	3.67	0.58	5.00	1.00	ns	4.33	4.48	1.89	1–9	0.13	0.453	0.169	−0.123	0.337
	2016	4.33	0.58	5.00	0.00	ns	4.67	4.49	1.47	1–9	0.10	0.064	0.172	−0.525	0.341
*tnm*	2014	3.00	0.00	5.67	0.58	Yes *P* < 0.05	4.33	3.84	1.12	1—7	0.08	0.416	0.187	−0.144	0.373
	2015	2.33	0.58	5.33	1.15	Yes *P* < 0.05	3.83	4.16	1.32	1–7	0.09	0.070	0.174	0.995	0.346
	2016	2.33	0.58	4.67	0.58	Yes *P* < 0.05	3.50	3.78	1.10	1–7	0.08	0.548	0.168	−0.612	0.335

*Variation between the mapping population parents presented in the form of means and standard deviations (SD) (n = 3) and the mid-parent value. The significance of differences between means was inferred using the Wilcoxon test. Variation across the mapping population presented as mean, SD, range, standard error (SE), and normality (skewness and kurtosis) (n = 213). Time of male flowering (tmf), time of female flowering (tff), and time of nut maturity (tnm) were rated from 1 = very early to 9 = very late. Dichogamy (dc) was rated from 1 = very protandrous to 9 = very protogynous.*

### Phenotypic Data of the Mapping Population

**FIGURE 1:**
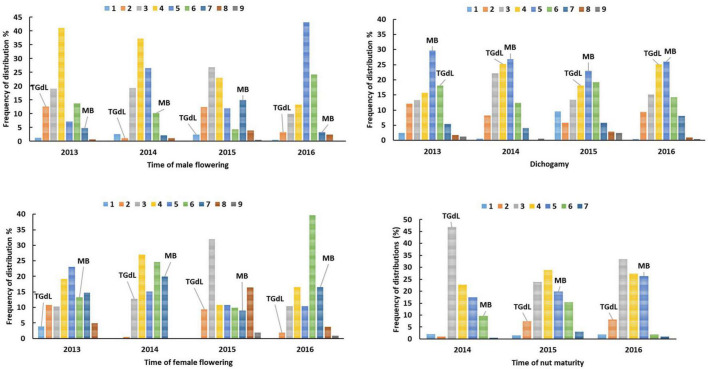
Frequency distribution plot for the mapping population with respect to time of male flowering (*tmf*), time of female flowering (*tff*), dichogamy (*dc*) and time of nut maturity (*tnm*) over four seasons. Data are grouped in classes from 1 = very early to 9 = very late. For dichogamy (*dc*), data are grouped in classes from 1 = very protandrous to 9 = very protogynous (5 = homogamous). The positions (means of three individuals) of the parents TGdL and MB are shown in each histogram.

Across the 213 individuals of the mapping population, flowering was initiated between late December/early January and continued to late February/early March. Nut fall began in late July and continued until mid-September (Supplementary Table 1). The *tmf* scores largely fell between those of the parents, with only a few examples of transgressive segregation (negative for two plants and positive for one, in 2013; positive in 2014 and 2015, respectively, for two and nine plants; positive for one plant and negative for five plants in 2016, Figure 1). For *tff*, the population mean consistently laid above the mid-parent value. Positive transgressive segregation was associated with ten individuals in both 2013 and 2016, and with 39 in 2015; however, in 2014, only one negative transgression was detected. The mapping population mean *dc* was intermediate between the parental values, and the distribution was normal. There was a substantial level of transgressive segregation in each year, affecting from 25.8% of the population in 2014 to 52.1% in 2013 (Figure 1). On the contrary, there was little evidence of transgressive segregation for *tnm* across the mapping population.

Inter-trait correlations both within and between years are shown in Table 2. The correlation across years within a trait was consistently positive and highly significant (*P* < 0.01): for *tmf*, the levels ranged from 0.81 to 0.87, for *tff* from 0.77 to 0.89, for *dc* from 0.71 to 0.76, for *tnm* from 0.48 to 0.67, and for *tlb* from 0.78 to 0.86 (*tlb* data from Torello Marinoni et al., 2018).

**TABLE 2:** Spearman correlation coefficients across years between each of the five traits: time of male flowering (*tmf*), time of female flowering (*tff*), dichogamy (*dc*), time of nut maturity (*tnm*), time of leaf budburst (*tlb*) and year of detection (*n* = 213).

	tmf_2013	tmf_2014	tmf_2015	tmf_2016	tff_2013	tff_2014	tff_2015	tff_2016	dc_2013	dc_2014	dc_2015	dc_2016	tnm_2014	tnm_2015	tnm_2016	tlb_2013	tlb_2014	tlb_2015	tlb_2016
tmf_2013	1	0.825**	0.811**	0.854**	0.440**	0.402**	0.432**	0.436**	0.261**	0.258**	0.238**	0.287**	0.294**	0.215**	0.215**	0.376**	0.463**	0.470**	0.506**
tmf_2014		1	0.849**	0.838**	0.431**	0.448**	0.405**	0.385**	0.133	0.345**	0.291**	0.296**	0.243**	0.202**	0.174*	0.322**	0.393**	0.458**	0.448**
tmf_2015			1	0.869**	0.452**	0.450**	0.433**	0.379**	0.141	0.243**	0.385**	0.312**	0.250**	0.228**	0.208**	0.311**	0.364**	0.426**	0.462**
tmf_2016				1	0.448**	0.412**	0.388**	0.401**	0.182*	0.269**	0.342**	0.408**	0.228**	0.209**	0.222**	0.357**	0.407**	0.427**	0.492**
tff_2013					1	0.824**	0.889**	0.845**	−0.709**	−0.476**	−0.497**	−0.459**	0.321**	0.325**	0.347**	0.666**	0.705**	0.671**	0.759**
tff_2014						1	0.852**	0.771**	−0.563**	−0.643**	−0.460**	−0.392**	0.335**	0.295**	0.348**	0.591**	0.609**	0.621**	0.702**
tff_2015							1	0.819**	−0.598**	−0.521**	−0.622**	−0.472**	0.314**	0.277**	0.361**	0.633**	0.673**	0.667**	0.749**
tff_2016								1	−0.570**	−0.484**	−0.492**	−0.626**	0.298**	0.259**	0.333**	0.658**	0.676**	0.640**	0.733**
dc_2013									1	0.711**	0.738**	0.745**	−0.099	−0.176*	−0.199*	−0.448**	−0.435**	−0.360**	−0.444**
dc_2014										1	0.722**	0.712**	−0.150*	−0.103	−0.224**	−0.411**	−0.348**	−0.303**	−0.386**
dc_2015											1	0.756**	−0.058	−0.053	−0.146*	−0.372**	−0.359**	−0.291**	−0.343**
dc_2016												1	−0.034	−0.068	−0.129	−0.374**	−0.334**	−0.274**	−0.314**
tnm_2014													1	0.478**	0.518**	0.297**	0.367**	0.267**	0.362**
tnm_2015														1	0.666**	0.224**	0.251**	0.280**	0.343**
tnm_2016															1	0.248**	0.300**	0.315**	0.395**
tlb_2013																1	0.830**	0.778**	0800**
tlb_2014																	1	0.843**	0.858**
tlb_2015																		1	0.860**
tlb_2016																			1

The tlb data are taken from Torello Marinoni et al. (2018). *Correlation is significant at 0.05 level (two-tailed). **Correlation is significant at 0.01 level (two-tailed).

The correlations between different traits were significant: *tmf* was positively correlated with each of the remaining traits, with values ranging from 0.38 to 0.45 for *tff*, from 0.22 to 0.45 for *dc*, from 0.29 to 0.51 for *tlb*, and from 0.15 to 0.28 for *tnm*. The *tff* was also positively correlated with both *tlb* (coefficients ranging from 0.59 to 0.73) and *tnm* (0.26–0.36), but was negatively correlated with *dc* (from −0.46 to −0.71). The *tlb* was positively correlated with *tnm* (from 0.22 to 0.39) and negatively with *dc* (from −0.27 to −0.45). The only trait combination showing any evidence of any correlation within a year was *tnm vs dc*, in both 2015 and 2016.

### Quantitative Trait Loci Analysis and Potential Candidate Genes Underlying Variation in Phenology

**FIGURE 2:**
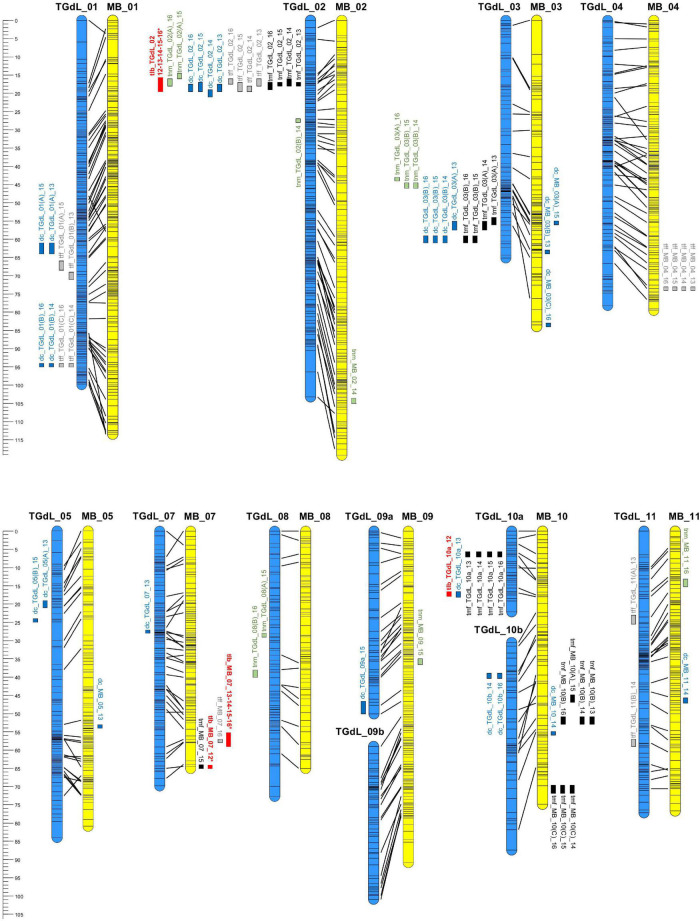
Linkage maps derived from TGdL (female parent of the mapping population) and MB (male parent). TGdL LGs are shown in blue on the left and MB ones in yellow on the right. The maps have been aligned based on shared scaffolds. The locations of the *tlb* QTL are shown in red. LGs not harboring any QTL have not been included. The ruler on the left represents the physical length of the LGs, while QTL locations are indicated in cM.

In all, 71 QTLs (49 on the TGdL map and 22 on the MB map) were identified. The loci were distributed across ten of the eleven LGs (only LG06 lacked at least one QTL). In all, 21 of the QTLs accounted for at least 10% of the phenotypic variance (PV) and are here indicated as “major” QTLs. The QTL associated with both the highest LOD (41.1) and the highest proportion of the PV (55.2%) was *tff_TGdL_02* (Table 3). Overall, among the major QTLs, 13 were detected in at least two of the seasons (stable-major QTLs), while three were specific to 2013/14, two were specific to 2014/15, and three were specific to 2015/16. Four QTLs were classified as minor in one of the seasons but as major in the other seasons (stable QTLs). The map locations of these QTLs are given in Figure 2. LG02 was found to harbor 16 QTLs (15 on TGdL, one on MB), while 15 QTLs mapped to LG10 (seven on TGdL map, eight on MB map) and 14 to LG03 (11 on TGdL map, and three on MB map).

**TABLE 3:** Time of male flowering (*tmf*), time of female flowering (*tff*), dichogamy (*dc*), and time of nut maturity (*tnm*) QTL identified using either the TGdL or the MB linkage map.

Trait	Parental	LG	QTL-harboring genomic regions	QTL season	Interval (cM)	Locus (marker)	Marker location (cM)	LOD	PV	Additive
*tmf*	TGdL	02	tmf_TGdL_02_13	2012/2013	16.0–19.0	04269_19964	17.6	6.3	9.5	−0.86
*tmf*	TGdL	02	tmf_TGdL_02_14	2013/2014	16.0–19.0	00690_17369	17.2	5.2	7.3	+ 0.64
*tmf*	TGdL	02	tmf_TGdL_02_15	2014/2015	16.0–19.0	04269_19964	17.6	8.0	9.8	−1.15
*tmf*	TGdL	02	tmf_TGdL_02_16	2015/2016	16.0–19.0	13518_3809	17.8	8.8	10.1	+ 0.79
*tmf*	TGdL	03	tmf_TGdL_03 (A)_13	2012/2013	54.0–57.5	03473_15811	55.0	5.8	8.7	−0.84
*tmf*	TGdL	03	tmf_TGdL_03 (A)_14	2013/2014	54.0–57.5	00313_57590	56.3	8.3	12.1	−0.84
*tmf*	TGdL	03	tmf_TGdL_03 (B)_15	2014/2015	59.0–61.0	18606_3110	60.5	6.7	8.1	−1.05
*tmf*	TGdL	03	tmf_TGdL_03 (B)_16	2015/2016	59.0–61.0	18606_3110	60.5	10.4	12.3	−0.87
*tmf*	TGdL	10a	tmf_TGdL_10a_13	2012/2013	5.6–7.0	00867_17261	6.4	12.5	20.7	+ 1.32
*tmf*	TGdL	10a	tmf_TGdL_10a_14	2013/2014	5.6–7.0	00867_17261	6.4	10.2	15.2	+ 0.96
*tmf*	TGdL	10a	tmf_TGdL_10a_15	2014/2015	5.6–7.0	00867_17261	6.4	15.8	21.3	+ 1.74
*tmf*	TGdL	10a	tmf_TGdL_10a_16	2015/2016	5.6–7.0	00867_17261	6.4	3.9	4.2	+ 0.65
*tmf*	MB	07	tmf_MB_07_15	2014/2015	64.0–65.0	00002_249778	64.5	3.1	5.8	+ 0.88
*tmf*	MB	10	tmf_MB_10 (A)_15	2014/2015	45.0–47.0	12683_1639	45.2	5.9	11.3	-1.35
*tmf*	MB	10	tmf_MB_10 (B)_13	2012/2013	50.5–53.0	05422_8180	51.3	3.5	9.0	+ 0.84
*tmf*	MB	10	tmf_MB_10 (B)_14	2013/2014	50.5–53.0	05422_8180	51.3	7.0	15.0	+ 1.11
*tmf*	MB	10	tmf_MB_10 (B)_16	2015/2016	50.5–53.0	05422_8180	51.3	7.7	15.3	+ 1.14
*tmf*	MB	10	tmf_MB_10 (C)_14	2013/2014	69.5–72.0	00231_56086	69.8	3.9	8.1	−0.84
*tmf*	MB	10	tmf_MB_10 (C)_15	2014/2015	69.5–72.0	00231_56086	69.8	3.8	7.0	−1.09
*tmf*	MB	10	tmf_MB_10 (C)_16	2015/2016	69.5–72.0	00231_56086	69.8	5.0	9.7	−0.92
*tff*	TGdL	01	tff_TGdL_01 (A)_15	2014/2015	66.0–68.5	10819_8333	67.1	6.0	5.5	−0.99
*tff*	TGdL	01	tff_TGdL_01 (B)_13	2012/2013	69.0–71.0	00654_47187	70.1	7.3	6.4	−0.92
*tff*	TGdL	01	tff_TGdL_01 (C)_14	2013/2014	94.0–95.0	00697_12571	94.1	3.5	4.0	−0.55
*tff*	TGdL	01	tff_TGdL_01 (C)_16	2015/2016	94.0–95.0	00697_12571	94.1	8.3	9.2	−0.88
*tff*	TGdL	02	tff_TGdL_02_13	2012/2013	16.0–19.5	00690_17369	17.2	41.1	55.2	+ 2.69
*tff*	TGdL	02	tff_TGdL_02_14	2013/2014	16.0–19.5	09783_7017	17.3	28.9	44.0	+ 1.83
*tff*	TGdL	02	tff_TGdL_02_15	2014/2015	16.0–19.5	00998_24907	18.6	40.0	55.1	+ 3.13
*tff*	TGdL	02	tff_TGdL_02_16	2015/2016	16.0–19.5	AJ417975b-LG2	19.2	30.4	43.7	+ 1.91
*tff*	TGdL	11	tff_TGdL_11 (A)_13	2012/2013	23.0–25.5	00472_27245	24.9	3.5	3.0	−0.63
*tff*	TGdL	11	tff_TGdL_11 (B)_14	2013/2014	57.0–59.0	00056_103944	58.1	4.0	4.5	−0.59
*tff*	MB	04	tff_MB_04_13	2012/2013	73.0–74.0	07153_8062	73.5	2.7	5.9	+ 0.88
*tff*	MB	04	tff_MB_04_14	2013/2014	73.0–74.0	07153_8062	73.5	3.3	6.9	+ 0.72
*tff*	MB	04	tff_MB_04_15	2014/2015	73.0–74.0	07153_8062	73.5	3.7	7.7	+ 1.17
*tff*	MB	04	tff_MB_04_16	2015/2016	73.0–74.0	07153_8062	73.5	3.95	7.8	+ 0.81
*tff*	MB	07	tff_MB_07_16	2015/2016	57.0–58.0	00932_5057	57.8	3.55	6.9	−0.77
dc	TGdL	01	dc_TGdL_01 (A)_13	2012/2013	61.0–64.0	04564_10133	62.4	6.3	6.8	−0.87
dc	TGdL	01	dc_TGdL_01 (A)_15	2014/2015	61.0–64.0	04564_10133	62.4	7.3	7.1	−1.03
dc	TGdL	01	dc_TGdL_01 (B)_14	2013/2014	94.0–95.0	00697_12571	94.1	3.2	4.3	+ 0.56
dc	TGdL	01	dc_TGdL_01 (B)_16	2015/2016	94.0–95.0	00697_12571	94.1	6.3	7.9	+0.84
dc	TGdL	02	dc_TGdL_02_13	2012/2013	17.0–21.0	00998_24907	18.6	21.4	29.1	−0.77
dc	TGdL	02	dc_TGdL_02_14	2013/2014	17.0–21.0	00145_43741	20.3	11.7	17.1	+ 1.11
dc	TGdL	02	dc_TGdL_02_15	2014/2015	17.0–21.0	AJ417975b-LG2	19.2	19.7	22.1	−1.80
dc	TGdL	02	dc_TGdL_02_16	2015/2016	17.0–21.0	00998_24907	18.6	10.2	13.3	−1.08
dc	TGdL	03	dc_TGdL_03 (A)_13	2012/2013	55.0–57.5	00313_57590	56.3	6.4	7.0	−0.89
dc	TGdL	03	dc_TGdL_03 (B)_14	2013/2014	59.0–61.0	18606_3110	60.5	6.9	9.5	−0.84
dc	TGdL	03	dc_TGdL_03 (B)_15	2014/2015	59.0–61.0	18606_3110	60.5	5.5	5.3	−0.88
dc	TGdL	03	dc_TGdL_03 (B)_16	2015/2016	59.0–61.0	18606_3110	60.5	6.2	7.7	−0.83
dc	TGdL	05	dc_TGdL_05 (A)_13	2012/2013	19.0–21.0	04009_17073	20.1	4.0	4.2	+ 0.69
dc	TGdL	05	dc_TGdL_05 (B)_15	2014/2015	24.0–25.0	00977_2263	24.3	5.2	4.9	+0.85
dc	TGdL	07	dc_TGdL_07_13	2012/2013	27.0–28.0	03819_15291	27.7	3.7	3.8	+ 0.65
dc	TGdL	09a	dc_TGdL_09a_15	2014/2015	46.6–50.1	09102_6077	47.8	15.4	16.5	+1.58
dc	TGdL	10a	dc_TGdL_10a_13	2012/2013	16.6–18.1	00361_58830	17.0	7.6	8.4	+ 0.98
dc	TGdL	10b	dc_TGdL_10b_14	2013/2014	8.4–9.9	04561_2721	8.8	6.0	8.1	+0.80
dc	TGdL	10b	dc_TGdL_10b_16	2015/2016	8.4–9.9	04561_2721	8.8	7.8	9.8	+ 0.96
dc	MB	03	dc_MB_03 (A)_15	2014/2015	55.0–56.0	16307_870	55.6	3.7	7.9	−1.06
dc	MB	03	dc_MB_03 (B)_13	2012/2013	63.0–64.0	01620_24360	63.8	2.6	6.6	−0.85
dc	MB	03	dc_MB_03 (C)_16	2015/2016	83.0–84.0	03721_8281	83.5	2.6	5.6	+ 0.71
dc	MB	05	dc_MB_05_13	2012/2013	53.0–54.0	11366_5928	53.4	3.1	7.8	+0.92
dc	MB	10	dc_MB_10_14	2013/2014	54.9–55.9	05502_4903	55.6	3.1	6.6	−0.68
dc	MB	11	dc_MB_11_14	2013/2014	45.5–47.0	00238_47928	46.3	3.7	7.9	+ 0.75
*tnm*	TGdL	02	tnm_TGdL_02 (A)_15	2014/2015	14.5–18.0	02660_24304	15.1	4.4	8.4	−0.77
*tnm*	TGdL	02	tnm_TGdL_02 (A)_16	2015/2016	14.5–18.0	04269_19964	17.6	5.8	10.6	−0.72
*tnm*	TGdL	02	tnm_TGdL_02 (B)_14	2013/2014	27.0–28.0	00141_82611	27.8	6.3	11.6	+ 0.76
*tnm*	TGdL	03	tnm_TGdL_03 (A)_16	2015/2016	43.0–44.0	00267_1457	43.6	3.0	5.4	−0.51
*tnm*	TGdL	03	tnm_TGdL_03 (B)_14	2013/2014	44.5–46.0	13395_3344	45.2	7.0	13.0	+ 0.81
*tnm*	TGdL	03	tnm_TGdL_03 (B)_15	2014/2015	44.5–46.0	13395_3344	45.2	3.5	6.7	+ 0.69
*tnm*	TGdL	08	tnm_TGdL_08 (A)_15	2014/2015	28.0–29.0	05647_13801	28.8	3.4	6.4	−0.67
*tnm*	TGdL	08	tnm_TGdL_08 (B)_16	2015/2016	38.0–40.0	12233_7197	38.7	3.1	5.6	−0.53
*tnm*	MB	02	tnm_MB_02_14	2013/2014	103.5–105.0	17280_1649	104.1	4.7	9.8	+ 0.70
*tnm*	MB	9	tnm_MB_09_15	2014/2015	35.0–36.5	05878_11879	35.8	3.2	7.0	+ 0.70
*tnm*	MB	11	tnm_MB_11_16	2015/2016	13.2–15.2	10305_7931	14.6	4.1	8.6	−0.65

*The table records for each QTL, the trait, the map parental where the QTL was detected, Linkage Group (LG), QTL name and season of detection, the interval of the QTL, the closest linked marker (Locus) and its map position in cM, the estimated LOD at the QTL peak (LOD), the PV explained (PV), and the contribution of each parent (Additive).*

Many of the QTLs were clustered with one another, as expected given the extensive degree of inter-trait correlation (Table 2). The major cluster on TGdL_02 consisted of mainly major QTL governing all of the traits. Two year-specific clusters were identified on TGdL_03 associated with *tmf* and *dc*, one comprising QTLs detected in both 2012/13 and 2013/14, and the other QTLs expressed in both 2014/15 and 2015/16. A cluster on TGdL_01 harbored minor QTLs for *dc* and *tff*, while three smaller clusters involving *tlb* (Torello Marinoni et al., 2018) included minor QTLs underlying *dc* (on TGdL_10a), *tmf*, and *tff* (in two distinct clusters at the bottom of MB_07).

#### Time of Male Flowering

Of the 20 QTLs identified as contributing to *tmf*, 12 were mapped on the TGdL map and eight on the MB map (Table 3), altogether 477 genes. With respect to the former set of QTLs, four genomic regions were identified, one on each of TGdL_02 and 10a, and two on TGdL_03. The LG10a QTL, linked to marker AJ_00867_17261, was responsible for 15–21% of the PV in the first three seasons (with LOD peak values of 10–16 and associated with an additive effect of 0.96–1.74 classes out of the nine detected), but only 4% in the fourth season (maximum LOD 3.90 and 0.65 additive value). The LG02 QTLs were detected in all four seasons and explained 7–10% of the PV, while the two regions on LG03 together accounted for 8–12% of the PV. The four regions together harbored 383 genes, of which six are implicated in control over aspects of flowering (Supplementary Table 2). In detail, *tmf_TGdL_02* carried 194 genes (3.484 – 5.116 Mb), *tmf_TGdL_10a* carried 155 genes (22.364–23.894 Mb), and *tmf_TGdL_10b* carried 34 genes (7.310 – 7.611 Mb). Of the four genomic regions identified in the MB map, three mapped to LG10; two of these harbored a QTL expressed in three of the four seasons, accounting for, respectively, 9–15% and 7–10% of the PV (with LOD peak values of 3.5–7.7 and 3.8–5.0, respectively, and both associated with additive effects of 0.8–1.1). A further QTL, responsible for 11% of the PV, was detected only in the 2014/15 season. The four regions together housed 94 genes, of which two are implicated in control over aspects of flowering (Supplementary Table 2). In detail, *tmf_MB_07* carried 42 genes (0.360–0.768 Mb) and *tmf_MB_10* carried 52 genes (3.353–3.774 Mb; 5.952–6.258 Mb).

#### Time of Female Flowering

Of the 15 *tff* QTLs detected, ten were located on the TGdL map and five on the MB map (Table 3), carrying 813 genes. The former set mapped within six genomic regions, distributed across LG01 (three regions), LG02, and LG11 (two regions). The largest effect QTL mapped between 17.2 and 19.2 cM along LG02, was detected in every season and explained 44–55% of the PV (with LOD peak values of 29–41 and associated with an additive effect of 1.8–3.1). The six genomic regions together harbored 731 genes, 19 of which are implicated in control over aspects of flowering (Supplementary Table 2). In detail, *tff_TGdL_01* carried 205 genes (33.032–34.473 Mb; 33.840–50.216 Mb), *tff_TGdL_02* carried 187 genes (2.743–5.038 Mb), *tff_TGdL_11 (A)* carried 257 genes (23.371–26.613 Mb), and *tff_TGdL_11 (B)* carried 82 genes (2.940 – 3.648 Mb). The loci located using the MB map were placed on two LGs. The one on LG04 was expressed in each year, but only explained 6–8% of the PV (maximum LOD 3.0–3.2 and 0.7–1.2 additive effect). The two regions housed 82 genes, but only one of these is implicated in control over aspects of flowering (Supplementary Table 2). In detail, *tff_MB_04* carried 45 genes (36.031 – 36.381 Mb) and *tff_MB_07* carried 37 genes (1.553 - 1.877 Mb).

#### Dichogamy

Of the 25 *dc* QTLs, 19 were located using the TGdL map and six the MB map (Table 3), carrying 1,688 genes. With respect to the former set, eleven genomic regions, distributed over eight LGs, were identified, including two regions each on LG01, LG03, LG05, and one region each on LG02, LG07, LG09a, LG10a, and LG10b. The largest effect QTL, which was detected in each of the four seasons, laid between 18.6 and 20.3 cM of LG02 and explained 13–29% of the PV (10–21 LOD peak values, 0.8–1.8 days of additive effect). A major QTL on LG09a (responsible for 16.5% of the PV) was detected only in the 2014/15 season. A minor QTL, explaining 5–9% in three of the 4 years was identified on LG03, as were loci expressed in 2 of the years on both LG01 and LG10b. The eleven regions housed 1,345 genes, of which 18 are implicated in potential control over dichogamy (Supplementary Table 2). In detail, *dc_TGdL_01 (A)* carried 108 genes (31.054 – 32.516 Mb), *dc_TGdL_01 (B)* carried 26 genes (49.781–50.216 Mb), *dc_TGdL_02* carried 233 genes (3.884–6.049 Mb), *dc_TGdL_05 (A)* carried 70 genes (31.646 – 32.384 Mb), *dc_TGdL_05 (B)* carried 18 genes (30.843 – 30.987 Mb), *dc_TGdL_07* carried 262 genes (13.173 – 20.404 Mb), *dc_TGdL_09a* carried 481 genes (22.804 – 29.178 Mb), *dc_TGdL_10a* carried 14 genes (19.215 – 19.364 Mb), and *dc_TGdL_10b* carried 133 genes (9.400 – 12.937 Mb). The six regions located using the MB map harbored only minor QTLs, each of which was only detected in a single season; these regions mapped to LG03 (three QTLs), LG05, LG10, and LG11, and housed 343 genes, of which four were associated with potential control over dichogamy (Supplementary Table 2). In detail, *dc_MB_05* carried 193 genes (15.072 – 20.183 Mb) and *dc_MB_10* carried 150 genes (2.692 – 3.873 Mb).

#### Time of Nut Maturity

Of the 11 *tnm* QTLs identified, eight were located using the TGdL map and three the MB map (Table 3), carrying 816 genes. The former fell within LG02, LG03, and LG08, with each LG housing two regions. Major QTLs mapped to the two regions on LG02, each accounting for 11–12% of the PV (maximum LOD 5.8–6.3; additive value 0.7–0.8). A QTL mapping to 45.2 cM along LG03 explained 7–13% of the PV in two of the four seasons. Together, the six regions harbored 507 genes, of which five were implicated in control over seed development (Supplementary Table 2). In detail, *tnm_TGdL_02 (A)* carried 237 genes (2.825–4.915 Mb), *tnm_TGdL_02 (B)* carried 67 genes (8.660–9.716 Mb), *tnm_TGdL_08 (A)* carried 91 genes (6.777–8.646 Mb), and *tnm_TGdL_08 (B)* carried 112 genes (13.405– 15.761 Mb). Using the MB map, a minor QTL was mapped on each of the LG02, LG09, and LG11; all of them were only expressed in a single season. The three regions together housed 309 genes, of which just two genes were associated with control over seed development (Supplementary Table 2). In detail, *tnm_MB_02* carried 88 genes (44.867–46.099 Mb), *tnm_MB_09* carried 64 genes (26.813–27.570 Mb), and *tnm_MB_11* carried 157 genes (26.925–28.570 Mb).

## Discussion

The present analysis was based on patterns of segregation for key phenological traits among progeny bred from a cross between two disparate parents: one of these (TGdL) is adapted to the climatic conditions prevalent in NW Italy, where it flowers early and its nuts mature early, whereas the other (MB), which was adapted to a rather cooler environment (Germany–East France), flowers later and its nuts are harvested later (Črepinšek et al., 2012). Exploiting a linkage map built from several hundred molecular markers (SNP and SSR markers) led to the recognition of a number of genomic regions harboring genes influencing the timing of both male and female flowering, dichogamy, and nut maturity. In several of these regions, the location of two or more QTLs responsible either for different traits (reflecting a set of tightly linked loci or, more likely, a single pleiotropic locus) and/or for the same trait across seasons overlapped. As an example, the major cluster on TGdL_02 consisted of mainly major QTLs governing all of the traits; this is the same region where Torello Marinoni et al. (2018) mapped a major QTL controlling leaf budburst trait.

### Phenotypic Variation

The variation for the various traits was typically not normally distributed, and furthermore, was not constant from 1 year to the next; the latter behavior reflects the major influence of the climate (particularly temperature) on these traits. The dates of *tmf* and *tff* in the progeny were similar over the years but the influence of temperature on the number of individuals in each class of distributions is evident. The effect of temperature on phenophase timing has been reported for numerous trees (Howe et al., 2003; Cooke et al., 2012; Ghelardini et al., 2014), including fruit tree species such as hazelnut (Črepinšek et al., 2012). As also noted by Črepinšek et al. (2012), the length of the period over which female flowering in hazelnut takes place can range from under one to over 10 weeks, depending on mean air temperature; early maturing cultivars such as TGdL are particularly labile in this respect. These authors also suggest that male and female flowers respond more strongly to increased air temperature than leafing.

As a consequence, the extent of dichogamy too is influenced jointly by genotype and climatic conditions (Bastias and Grau, 2005; Črepinšek et al., 2012); the dichogamy type of certain hazelnut cultivars, including MB, can be affected by the climate (Turcu et al., 2001; Črepinšek et al., 2012). Transgressive segregation in both directions was observed for each of the traits (Figure 1). This even included *dc*, even though the two parental lines did not differ significantly from one another for this trait. Trait transgression typically arises as a result of the inheritance of novel combinations of distinct alleles present in each parent (de Vicente and Tanksley, 1993). For each of the traits, there was a high positive correlation (*P* < 0.01) between the performance of given progeny in the various seasons, while there were also extensive, mostly positive correlations between pairs of traits (the exception was the negative correlation between *dc* and both *tff* and *tlb*). The trait which was least well correlated with others was *tnm*.

### The Mode of Inheritance of the Phenological Traits

The genetic basis of phenology is complex as a result of the numerous physiological pathways that are involved. Until now, the only phenological trait of hazelnut to be genetically analyzed has been *tlb* (Torello Marinoni et al., 2018). The present research has extended the knowledge based on phenological traits by revealing that several QTLs underlie variation for each of the traits investigated. While most of these QTLs individually explained less than 10% of the PV, about a quarter of them proved to be stable over years and some were responsible for quite a high proportion of the PV; these latter loci in principle could be targeted for marker-assisted selection. The most substantial *tff* QTL, which accounted for as much as 55% of the PV, mapped between 17 and 19 cM on TGdL_02. Meanwhile, the largest effect *tmf* QTL mapped near one end (6.4 cM) of TGdL_10a was detected; the observation that this QTL was rather poorly expressed in one of the four seasons suggests that it is regulated by gene(s) which are responsive to environmental conditions.

Of particular note is the coincident map location of a number of the QTLs, which implies that a degree of pleiotropy underlies variation for these traits. The most striking example relates to the region of TGdL_02 lying between 17.2 and 27.8 cM, within which QTLs controlling *tff*, *tmf*, *dc*, and *tnm* were consistently mapped across years (Figure 2). The same region also harbors a QTL for *tlb* (Torello Marinoni et al., 2018). A useful marker for this cluster of QTLs is the microsatellite locus AJ417975b. A second important genomic region along TGdL_03 harbored QTsL involved in the determination of *tmf*, *dc*, and *tnm*; while *dc* and *tmf* QTLs both mapped in the segment between 55 and 60 cM, the *tnm* QTL was located in a somewhat less distal segment (43–45 cM). There was also a coincident location for *dc* and *tff* on TGdL_01; the association of these two traits is understandable, since dichogamy, is strongly influenced by climatic conditions (Črepinšek et al., 2012).

The genetic basis of flowering time in hazelnut remains poorly researched, in contrast to the situation in pear (*Pyrus communis*) (Gabay et al., 2018; Ntladi et al., 2018), apple (*Malus domestica*) (Allard et al., 2016), apricot (*Prunus armeniaca*) (Kitamura et al., 2018), and willow (*Salix* spp.) (Ghelardini et al., 2014). In both pear (Gabay et al., 2018) and apple (Allard et al., 2016), the location of some QTLs underlying variation in flowering time coincides with those associated with vegetative budburst, just as was the case with hazelnut. Similar pleiotropic effects observed in willow have been taken to imply that the determination of these phenological traits shares common components (Ghelardini et al., 2014), a conclusion which is not unexpected given that they are all strongly influenced by temperature and/or photoperiod.

### Candidate Genes Underlying Major Phenological Trait Quantitative Trait Loci

The number of genes present in the regions harboring *tmf, tff, dc*, and *tnm* QTLs were, respectively: 477 (383 in the TGdL and 94 in the MB maps), 813 (731 in the TGdL and 82 in the MB), 1,688 (1,345 in the TGdL and 193 in the MB), and 816 (507 in the TGdL and 309 in the MB). Some of these were expected to include genes involved in the control of dormancy, budburst, the switch from vegetative to reproductive growth, or the morphogenesis of flowers and seeds. Accordingly, many of them have been described in the literature as involved in processes related to flowering, at the molecular level (Supplementary Figure 1) and to specific plant ontology (PO) terms: *pollen-tube-cell (PO:0025195), plant sperm cell (PO:0000084), pollen (PO:0025281), inflorescence-meristem (PO:0000230), carpel (PO:0009030) stamen (PO:0009029)*, and *flower (PO:0009046).* Genes in the QTL intervals were thus discussed when showing a clear function/annotation related to flowering-like processes in a range of plant species, as inferred from literature (Supplementary Table 2).

#### Candidate Genes for Time of Male Flowering

Five genes appeared strongly related to flowering phenotype (Supplementary Table 2). Haze_17445, in *tmf_MB_07*, is a homolog of *TEM1*, which in *Arabidopsis thaliana* encodes a protein acting to delay flowering by inhibiting the production of both FT and the hormone gibberellic acid, until such time as the plant has matured beyond a particular growth stage (Castillejo and Pelaz, 2008; Matías-Hernández et al., 2014). Haze_04134, in *tmf_TGdL_02*, is a homolog of the *A. thaliana* gene *RFI2.* It encodes an E3 ubiquitin-protein ligase (Chen and Ni, 2006) known to negatively regulate the CONSTANS/FLOWERING LOCUS T (CO/FT) module, which is key to the promotion of flowering in response to photoperiod (Chen and Ni, 2006; Turck et al., 2008). The other three genes are homologs of genes involved in determining the timing of anthesis; Haze_21037, in *tmf_TGdL_10a*, a homolog of *MSP1* which encodes a leucine-rich repeat receptor protein kinase; Haze_17456, in *tmf_MB_07*, a homolog of *PP2AA2* (serine/threonine-protein phosphatase 2A 65 kDa regulatory subunit A), and Haze_20983, in *tmf_TGdL_10a*, a homolog of *CPL2* (RNA polymerase II C-terminal domain phosphatase-like 2) (Supplementary Table 2). In *A. thaliana*, these latter three genes are all involved in sporogenesis and the regulation of floral development through phosphorylation/dephosphorylation circuits (Nonomura et al., 2003; Ueda et al., 2008; Kataya et al., 2015). Other three genes are potential homologs of DPD1 (Tang et al., 2012), MBD9 (Peng et al., 2006) and APD1 (Luo et al., 2012).

#### Candidate Genes for Time of Female Flowering

A total of 16 genes strongly related to flowering were present in the genomic regions harboring *tff* QTLs (Supplementary Table 2). The first of these is Haze_16689, in *tff_TGdL_11 (A)*, a homolog of rice *HDR1* and an ortholog of *CO*, genes that regulate the photoperiod-dependent flowering pathway (Sun et al., 2016). The second is Haze_16712, in *tff_TGdL_11 (A)*, and a homolog of *SOC1* (SUPPRESSOR OF OVEREXPRESSION OF CONSTANS1) which encodes a transcription activator of LEAFY (Gregis et al., 2009). The third is Haze_15371, in *tff_TGdL_11 (B)*, a potential homolog of *A. thaliana AP2* which encodes a transcription factor activating the floral meristem (Krogan et al., 2012). Haze_15391, in *tff_TGdL_11 (B)*, is a strong candidate for *tff* given that the product of its homolog is probably involved in the auxin-mediated control of gynoecium patterning (Gremski et al., 2007). The five genes Haze_02311, in *tff_TGdL_01 (B)*, Haze_16714 and Haze_16827, in *tff_TGdL_11 (A)*, Haze_15445, in *tff_TGdL_11 (B)*, and Haze_17592, in *tff_MB_07*, are also homologs of relevant transcription factors in *A*. *thaliana*, namely *SRS3* (Kuusk et al., 2006), *MADS3* (Fernandez et al., 2013), *AHL18* (Xiao et al., 2009), *HHO5* (Moreau et al., 2016), *and KAN4* (Gao et al., 2010), respectively. The tenth potential candidate gene is Haze_04134, in *tff_TGdL_02*, which as noted above, is a homolog of *A. thaliana RFI2* (Chen and Ni, 2006). A group of six genes: Haze_02317, Haze_02318, in *tff_TGdL_01 (B*), and Haze_02223, in *tff_TGdL_01 (A)*, Haze_04095, in *tff_TGdL_02*, Haze_16862, in *tff_TGdL_11 (A)*, Haze_15376, in *tff_TGdL_11 (B)*, likely encoding proteins responsible for histone methylation/demethylation/acetylation, are also plausible as candidates, because it is known that the transcript level of the key genes *FT* and *FC* is regulated epigenetically (Jeong et al., 2015). These six genes are potential homologs of, respectively, *WDR5A* (Jiang et al., 2009), *SHL* (Lopez-Gonzalez et al., 2014), *TAF14B* (Bieluszewski et al., 2015), *MBD9* (Yaish et al., 2009), *ELF6* (Jeong et al., 2009) *and CLF* (Saleh et al., 2007). Other four genes are homologs of *MEE40* (Pagnussat et al., 2005), *QKY* (Fulton et al., 2010), *FLD* (Liu et al., 2007) and *NFD4* (Portereiko et al., 2006).

#### Candidate Genes for Dichogamy

Many species of plants have developed mechanisms that prevent self-pollination. Although the hazelnut typically exhibits sporophytic self-incompatibility, many cultivars are also dichogamous. Fourteen genes were related to the dichogamy trait were identified (Supplementary Table 2). The genes Haze_02127, in *dc_TGdL_01 (A*), Haze_04221, in *dc_TGdL_02*, Haze_11214, *Haze*_11325, Haze_11453, in *dc_TGdL_09a*, Haze_20664, in *dc_TGdL_10a*, Haze_13480, in *dc_MB_05*, and Haze_19677, in *dc_MB_10*, encode eight transcription factors involved in the determination of cell fate and organ development; these genes were homologs of, respectively, *AMS* (Lou et al., 2014), *NFYC9* (Hou et al., 2014), *TCX2* (Sijacic et al., 2011), *SUP* (Hiratsu et al., 2002), *CO3* (Kim et al., 2008), *AP2-3* (Lee et al., 2007), *MADS1* (Poupin et al., 2007), and *OFP13* (Wang et al., 2011). A further set of candidate genes: Haze_04202, in *dc_TGdL_02*, Haze_18674, in *dc_TGdL_07*, Haze_11374, Haze_11526, and Haze_11258, in *dc_TGdL_09a*, potentially encode five regulators of flowering. Haze_11374 is a homolog of *CRY2*, the product of which is a major photoreceptor regulating *FT* and *FLC* (Endo et al., 2007). Haze_11526 is a homolog of *BLI*, the product of which controls cotyledon and leaf patterning by inhibiting premature differentiation (Schatlowski et al., 2010); in particular, this gene is required for the activation of *FLC* and is involved in the response to chilling. Haze_18674 is a homolog of *MED8*, the product of which is involved in the regulation of flowering time (Lalanne et al., 2004; Kidd et al., 2009). Haze_04202 is a homolog of *MIRO1*, the product of which is a mitochondrial GTPase required during gametogenesis (Sormo et al., 2011). Haze_11258 is a homolog of *LFR*, the product of which is a nuclear protein required for the formation of anthers and may be a key component of the genetic network regulating anther development (Wang et al., 2012). The final potential candidate is Haze_14427, in *dc_TGdL_05 (B)*, a homolog of *RPK2*, the product of which is an LRR receptor-like serine/threonine-protein kinase, involved in the regulation of anther development, including tapetum degradation during pollen maturation (Mizuno et al., 2007; Nodine et al., 2007). Other genes are potential homologs of *NFD4* (Portereiko et al., 2006), *CLPS3* (Xing et al., 2008), *HUA1* (Cheng et al., 2003), *EDA40* (Pagnussat et al., 2005), *CLO* (Liu et al., 2009), *RIE1* (Xu and Li, 2003), *JASON* (De Storme and Geelen, 2011), *LIS* (Voelz et al., 2012).

#### Candidate Genes for Time of Nut Maturity

In hazelnut, the ovary has yet to form at the time when the pistil is mature. The differentiation of several layers of ovary primordial cells occurs only after fertilization has been achieved. The identity of the genes that regulate ovary and ovule development in hazelnut is largely unknown.

Five genes potentially influencing seed development were placed within a genomic region harboring the *tnm* QTLs. These genes were Haze_03973, Haze_11440, Haze_04470, Haze_25121 and Haze_16946. Haze_03973, in *tnm_TGdL_02 (A)*, and Haze_11440, in *tnm_MB_09* are homologs of *ACS3* and *ACS1*, respectively. The product of *ACS3* is 1-aminocyclopropane-1-carboxylate synthase 3, while that of *ACS1* is 1-aminocyclopropane-1-carboxylate synthase; both of these enzymes catalyze 1-aminocyclopropane-1-carboxylic acid, a direct precursor of ethylene (Peng et al., 2005). In *A. thaliana*, the absence of this compound results in abnormal embryo morphogenesis and embryo lethality (Baud et al., 2003), while Cheng et al. (2019) have suggested that since the mature hazelnut kernel is rich in unsaturated fatty acids, its absence impairs the conversion of citrate into long-chain fatty acids, thereby compromising the development of the ovule. This is a very interesting finding since the occurrence of blank nuts in hazelnut is still poorly understood, but represents a serious problem to solve to avoid important losses of yields in orchards. Haze_04470, in *tnm_TGdL_02 (B)* is a homolog of *EIN4*, the product of which is a negative regulator of ethylene signaling (Hua et al., 1998). Haze_25121, in *tnm_TGdL_08 (B)* encodes a protein thought to be involved in the abscission of the mature nut (Liljegren et al., 2004); the gene is a homolog of *IND*, the product of which is a transcription factor involved in the differentiation of various cell types required for fruit dehiscence (Liljegren et al., 2004). Finally, Haze_16946, in *tnm_MB_11*, is a homolog of *AP2-2*, the product of which is a member of AP2/ERF family of transcription factors involved in the control of inflorescence architecture and floral meristem establishment (Dai et al., 2016). Other two genes are potential homologs of *GPA3* (Ren et al., 2014) and *IDL4* (Stenvik et al., 2008).

## Conclusion

The present study explores the genetic architecture of phenology traits in a progeny of *Corylus avellana.* The QTL mapping described here has identified for the first time (except for *tlb* trait) several major QTLs underlying phenological-related traits in hazelnut (time of male and female flowering, dichogamy, and nut maturity). Several regions were identified where many QTLs co-localized for different traits or for the same trait across years. The 26% of identified QTLs were very robust, stable, being therefore promising for the use in marker-assisted selection.

The search of genes along the scaffolds linked to the QTL of interest has shown some interesting match with orthologous genes involved in flowering and nut growth processes in other plant species. The presence of homologs of genes known to be involved in the determination of flowering time and seed development in the relevant genomic regions has provided some leads toward gaining an understanding of the genetic and molecular basis of these important traits.

The regions surrounding the inferred locations of the major QTLs harbor hundreds of genes, some of which will likely represent fruitful targets for future investigations. Resequencing of strong candidate genes could be used to reveal the extent of allelic variation present in phenotypically contrasting cultivars. The availability of a mapping population will be useful in narrowing the search for genuine candidate genes.

The need to develop genomic tools able to accelerate hazelnut breeding is increasing. The data reported here will make a contribution toward the formulation of a biotechnology-based strategy designed to efficiently select trees more likely to be able to adapt to a changing environment.

## Data Availability Statement

The raw data supporting the conclusions of this article will be made available by the authors, without undue reservation.

## Author Contributions

RB conceived and designed the experiments. NV and DTM performed field investigations and analyzed field data. DTM and EP performed QTL analysis. AA, VP, and ECG performed candidate genes and functional annotation analysis. DTM, NV, and EP wrote the manuscript. All authors contributed to the discussion, revised, and approved the final manuscript.

## Conflict of Interest

The authors declare that the research was conducted in the absence of any commercial or financial relationships that could be construed as a potential conflict of interest.

## Publisher’s Note

All claims expressed in this article are solely those of the authors and do not necessarily represent those of their affiliated organizations, or those of the publisher, the editors and the reviewers. Any product that may be evaluated in this article, or claim that may be made by its manufacturer, is not guaranteed or endorsed by the publisher.

## Footnotes

1
https://www.cavellanagenomeportal.com

2
https://www.ebi.ac.uk/ena/data/view/PRJEB31933

3
https://zenodo.org/record/4454484

4
https://www.ibm.com/

## Supplementary Material

Supplementary Figure 1 Candidate genes underlying variation in phenology in hazelnut. **(A)** A four-way Venn diagram illustrating overlap in the genes mapping within the regions harboring *tmf*, *tff*, *dc*, and *tnm* QTL. **(B)** Functional categorization of the 121 genes potentially underlying variation in *tmf*, *tff*, *dc*, and *tnm*. **(C)** Functional categorization of candidate genes mapping within regions harboring either *tmf*, *tff*, *dc*, or *tnm* QTL.

Supplementary Table 1 Dates of male (*tmf*) and female flowering (*tff*) and nut maturity (*tnm*): the data refer to the earliest and latest flowering and maturing segregants recorded over four seasons.

Supplementary Table 2 The gene content of the regions harboring time of male flowering (*tmf*), time of female flowering (*tff*), dichogamy (*dc*), and time of nut maturity (*tnm*) QTL. Hazelnut genes (Haze_Xx) were identified using the TGdL genome.gff annotation file (https://zenodo.org/record/4454484).
