# Supplementary figures and images for: Mapping the Genetic Regions Responsible for Key Phenology-Related Traits in the European Hazelnut
Source: Front Plant Sci. 2021 Dec 23;12:749394. doi: 10.3389/fpls.2021.749394 (PMC8733624; doi:10.3389/fpls.2021.749394)

**(A)**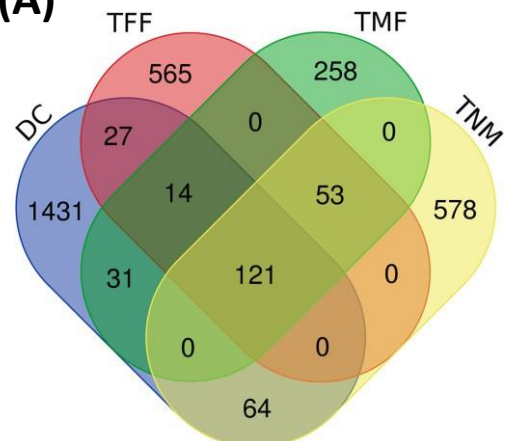**(C)**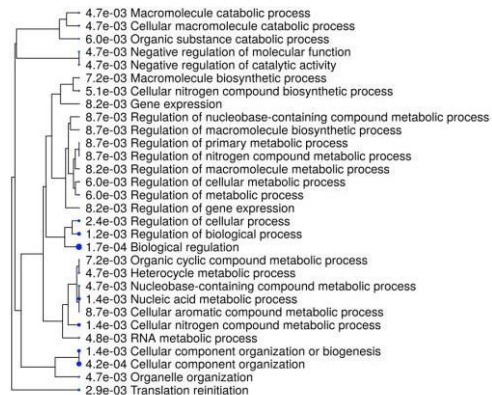**in common****(B)****TMF**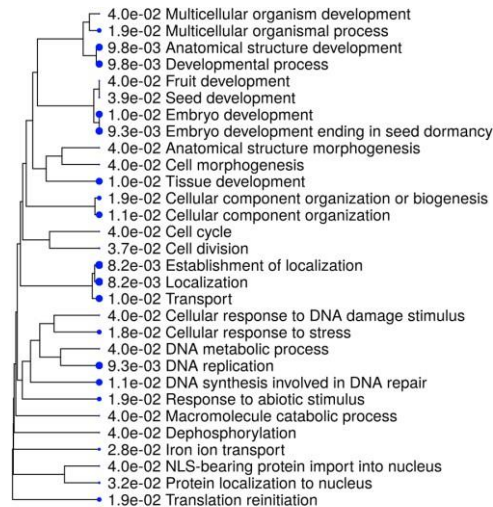**DC****TFF**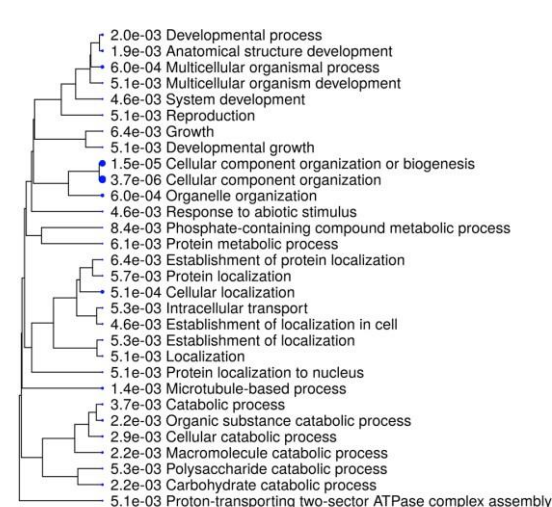**TNM**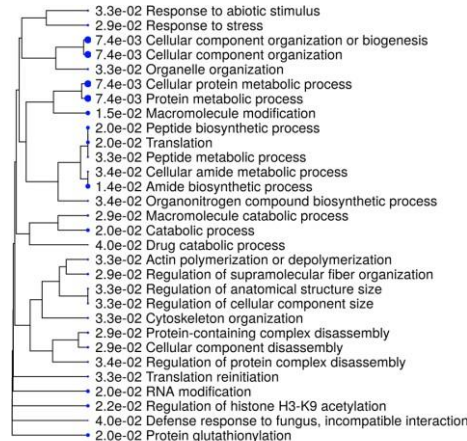

Supplement: Supplementary Figure 1 — Candidate genes underlying variation in phenology in hazelnut. (A) A four-way Venn diagram illustrating overlap in the genes mapping within the regions harboring tmf, tff, dc, and tnm QTL. (B) Functional categorization of the 121 genes potentially underlying variation in tmf, tff, dc, and tnm. (C) Functional categorization of candidate genes mapping within regions harboring either tmf, tff, dc, or tnm QTL. [file Image_1.pdf]
