# Supplementary material for: Mapping the Genetic Regions Responsible for Key Phenology-Related Traits in the European Hazelnut
Source: Front Plant Sci. 2021 Dec 23;12:749394. doi: 10.3389/fpls.2021.749394 (PMC8733624; doi:10.3389/fpls.2021.749394)
Supplement: Supplementary Table 1 — Dates of male (tmf) and female flowering (tff) and nut maturity (tnm): the data refer to the earliest and latest flowering and maturing segregants recorded over four seasons. [file Table_1.DOCX]

**Suppl S1.** Dates of male (*tmf*) and female flowering (*tff*) and nut maturity (*tnm*): the data refer to the earliest and latest flowering and maturing segregants recorded over four seasons. Dates of male flowering was assessed when 10% of catkins started to release pollen; female flowering was recorded when 10% of female flowers were receptive, date of nut maturity was determined when 10% of nuts was fallen to the ground.

|  |  | **Male and female flowering** | | **Nut Maturity** | |
| --- | --- | --- | --- | --- | --- |
| **Season** |  | **Date for earliest individuals** | **Date for latest individuals** | **Date for earliest individuals** | **Date for latest individuals** |
| **2012-13** |  | 02 January 2013 | 11 March 2013 | - | - |
| **2013-14** |  | 30 December 2013 | 5 March 2014 | 4 August 2014 | 15 September 2014 |
| **2014-15** |  | 22 December 2014 | 20 February 2015 | 30 July 2015 | 10 September 2015 |
| **2015-16** |  | 23 December 2015 | 25 February 2016 | 3 August 2016 | 14 September 2016 |
