# Supplementary material for: Mapping the Genetic Regions Responsible for Key Phenology-Related Traits in the European Hazelnut
Source: Front Plant Sci. 2021 Dec 23;12:749394. doi: 10.3389/fpls.2021.749394 (PMC8733624; doi:10.3389/fpls.2021.749394)
Supplement: Supplementary Table 2 — The gene content of the regions harboring time of male flowering (tmf), time of female flowering (tff), dichogamy (dc), and time of nut maturity (tnm) QTL. Hazelnut genes (Haze_Xx) were identified using the TGdL genome.gff annotation file (https://zenodo.org/record/4454484). [file Table_2.pdf]

Supplementary Table 2 | The gene content of the regions harboring time of male flowering (tmf), time of female flowering (tff), dichogamy (dc), and time of nut maturity (tnm) Qtl. Hazelnut genes (Haze_Xx) were identified using the TgdL genome.gff annotation file (https://zenodo.org/record/4454484).

Trait	Hazelnut Gene	TGdL genome coordinates	QTL	Position (cM)	Annotation	Function	Citation
Time of male flowering (*tmf*)	Haze_04092	chr2:4267342-4272051	TGdL_02	16.0-19.0 cM	Similar to DPD1: Exonuclease DPD12C chloroplastic/mitochondrial (Arabidopsis thaliana OX3D3702)	In pollen development this exonuclease is required for organelle DNA degradation	Tang et al. 2012
	Haze_04095	chr2:4285133-4289006	TGdL_02	16.0-19.0 cM	Similar to MBD9: Methyl-CpG-binding domain-containing protein 9 (Arabidopsis thaliana OX3D3702)	Probable histone acetyltransferase involved in flowering time	Peng et al. 2006
	Haze_04134	chr2:4664888-4673881	TGdL_02	16.0-19.0 cM	Similar to RFI2: E3 ubiquitin-protein ligase RFI2 (Arabidopsis thaliana OX3D3702)	Regulates negatively CO and FT in light-controlled pathway	Chen and Ni, 2006
	Haze_20983	chr10:22824772-22828138	TGdL_10a	5.6-7 cM	Similar to CPL2: RNA polymerase II C-terminal domain phosphatase-like 2 (Arabidopsis thaliana OX3D3702)	The dephosphorylation activity is required for male gametes fertility	Ueda et al. 2008
	Haze_21004	chr10:23009745-23017880	TGdL_10a	5.6-7 cM	Similar to APD1: E3 ubiquitin-protein ligase APD1 (Arabidopsis thaliana OX3D3702)	Involved in regulation in male gametogenesis	Luo et al. 2012
	Haze_21037	chr10:23332319-23339739	TGdL_10a	5.6-7 cM	Similar to MSP1: Leucine-rich repeat receptor protein kinase MSP1 (Oryza sativa subsp. japonica OX3D39947)	Receptor-like kinase with a putative role in male and female sporogenesis	Nomomura et al. 2003
	Haze_17445	chr7:514006-515999	MB_07	64.0-65.0 cM	Similar to TEM1: AP2/ERF and B3 domain-containing transcription repressor TEM1 (Arabidopsis thaliana OX3D3702)	Repressor of FT that directly binds the DNA	Castillejo and Pelaz, 2008
	Haze_17456	chr7:628080-641223	MB_07	64.0-65.0 cM	Similar to PP2AA2: Serine/threonine-protein phosphatase 2A 65 kDa regulatory subunit A beta isoform (Arabidopsis thaliana OX3D3702)	Subunit of a phosphatase involved in floral development	Kataya et al. 2015
	Haze_02223	chr1:33333922-33344314	TGdL_01 (A)	66.0-68.5 cM	Similar to TAF14B: Transcription initiation factor TFIID subunit 14b (Arabidopsis thaliana OX3D3702)	Controls histone acetylation levels; regulates negatively FT and positively FLC	Bieluszewski et al. 2015
	Haze_02311	chr1:34558621-34559454	TGdL_01 (B)	69.0-71.0 cM	Similar to SRS3: Protein SHI RELATED SEQUENCE 3 (Arabidopsis thaliana OX3D3702)	Transcription activator that influences gynoecium development	Kuusk et al. 2006

Time of female flowering (*tff*)	Haze_02317	chr1:34622852-34629808	TGdL_01 (B)	69.0-71.0 cM	Similar to WDR5A: COMPASS-like H3K4 histone methylase component WDR5A (Arabidopsis thaliana OX3D3702)	Delay flowering time through methylation mediated by COMPASS-like complexes	Jiang et al. 2009
	Haze_02318	chr1:34635109-34639253	TGdL_01 (B)	69.0-71.0 cM	Similar to SHL: Chromatin remodelling protein SHL (Arabidopsis thaliana OX3D3702)	Prevent histone acetylation, required for flowering and fertility	Lopez-Gonzalez et al. 2014
	Haze_03995	chr2:3494349-3498036	TGdL_02	16.0-19.5 cM	Similar to MEE40: Pentatricopeptide repeat-containing protein At3g537002C chloroplastic (Arabidopsis thaliana OX3D3702)	May be involved in female gametophyte development	Pagnussat et al. 2005
	Haze_04095	chr2:4285133-4289006	TGdL_02	16.0-19.5 cM	Similar to MBD9: Methyl-CpG-binding domain-containing protein 9 (Arabidopsis thaliana OX3D3702)	Probable histone acetyltransferase, involved in regulation of flowering time	Yaish et al. 2009
	Haze_04134	chr2:4664888-4673881	TGdL_02	16.0-19.5 cM	Similar to RFI2: E3 ubiquitin-protein ligase RFI2 (Arabidopsis thaliana OX3D3702)	Regulates negatively CO and FT in light-controlled pathway	Chen and Ni, 2006
	Haze_16637	chr11:23644037-23646200	TGdL_11 (A)	23.0-25.5 cM	Similar to QKY: Protein QUIRKY (Arabidopsis thaliana OX3D3702)	Regulates organ development through Ca 2+-dependent signaling and membrane trafficking	Fulton et al. 2010
	Haze_16689	chr11:24346664-24347564	TGdL_11 (A)	23.0-25.5 cM	Similar to HDR1: Protein HEADING DATE REPRESSOR 1 (Oryza sativa subsp. japonica OX3D39947)	Regulates the photoperiod-dependent pathway	Sun et al. 2016
	Haze_16712	chr11:24650276-24653381	TGdL_11 (A)	23.0-25.5 cM	Similar to SOC1: MADS-box protein SOC1 (Arabidopsis thaliana OX3D3702)	Transcription activator of LFY, it integrates different signals	Gregis et al. 2009
	Haze_16714	chr11:24659950-24667562	TGdL_11 (A)	23.0-25.5 cM	Similar to MADS3: Agamous-like MADS-box protein MADS3 (Vitis vinifera OX3D29760)	Probable transcription factor linked to flower development	Fernandez et al. 2013
	Haze_16741	chr11:25038324-25043965	TGdL_11 (A)	23.0-25.5 cM	Similar to FLD: Protein FLOWERING LOCUS D (Arabidopsis thaliana OX3D3702)	Probable histone demethylase that suppress FLC	Liu et al. 2007
	Haze_16752	chr11:25123082-25131662	TGdL_11 (A)	23.0-25.5 cM	Similar to NFD4: Protein NUCLEAR FUSION DEFECTIVE 4 (Arabidopsis thaliana OX3D3702)	Required for the fusion of the two polar nuclei for the diploid central cell nucleus formation.	Portereiko et al. 2006
	Haze_16827	chr11:25989814-25990734	TGdL_11 (A)	23.0-25.5 cM	Similar to AHL18: AT-hook motif nuclear-localized protein 18 (Arabidopsis thaliana OX3D3702)	Transcription factor that binds MARs, regulates flowering	Xiao et al. 2009
	Haze_16862	chr11:26485877-26496111	TGdL_11 (A)	23.0-25.5 cM	Similar to ELF6: Probable lysine-specific demethylase ELF6 (Arabidopsis thaliana OX3D3702)	Probable histone demethylase, repressor of FT	Jeong et al. 2009

	Haze_15371	chr11:2979559-2983019	TGdL_11 (B)	57.0-59.0 cM	Similar to AP2: Floral homeotic protein APETALA 2 (Arabidopsis thaliana OX3D3702)	Probable transcriptional activator required for meristem floral transition	Krogan et al. 2012
	Haze_15376	chr11:3035205-3049465	TGdL_11 (B)	57.0-59.0 cM	Similar to CLF: Histone-lysine N-methyltransferase CLF (Arabidopsis thaliana OX3D3702)	Polycomb group protein required for floral development by repressing AGAMOUS	Saleh et al. 2007
	Haze_15391	chr11:3154915-3155577	TGdL_11 (B)	57.0-59.0 cM	Similar to HEC1: Transcription factor HEC1 (Arabidopsis thaliana OX3D3702)	Required for the development of reproductive female structures	Gremski et al. 2007
	Haze_15445	chr11:3610787-3613366	TGdL_11 (B)	57.0-59.0 cM	Similar to HHO5: Transcription factor HHO5 (Arabidopsis thaliana OX3D3702)	Transcriptional repressor that regulates the flower development	Moreau et al. 2016
	Haze_17592	chr7:1829090-1832811	MB_07	57.0-58.0 cM	Similar to KAN4: Probable transcription factor KAN4 (Arabidopsis thaliana OX3D3702)	Required for flower integuments formations	Gao et al. 2010
Dichogamy (*dc*)	Haze_02127	chr1:32310780-32319418	TGdL_01 (A)	61.0-64.0 cM	Similar to AMS: Transcription factor ABORTED MICROSPORES (Arabidopsis thaliana OX3D3702)	Transcription factor required in tapetum development and male fertility	Lou et al. 2014
	Haze_03380	chr1:49990865-49993246	TGdL_01 (B)	94.0-95.0 cM	Similar to NFD4: Protein NUCLEAR FUSION DEFECTIVE 4 (Arabidopsis thaliana OX3D3702)	Required for the fusion of the two polar nuclei for the diploid central cell nucleus formation	Portereiko et al. 2006
	Haze_04202	chr2:5275881-5285574	TGdL_02	17.0-21.0 cM	Similar to MIRO1: Mitochondrial Rho GTPase 1 (Arabidopsis thaliana OX3D3702)	Mitochondrial GTPase required for maintenance of mitochondria during development of haploid male and female gametes	Sormo et al. 2011
	Haze_04221	chr2:5432511-5433299	TGdL_02	17.0-21.0 cM	Similar to NFYC9: Nuclear transcription factor Y subunit C-9 (Arabidopsis thaliana OX3D3702)	Transcriptional activator of photoperiodic and GA pathways	Hou et al. 2014
	Haze_14584	chr5:32289895-32295734	TGdL_05 (A)	19.0-21.0 cM	Similar to CLPS3: Protein CLP1 homolog (Arabidopsis thaliana OX3D3702)	Involved in gametophyte, embryo and postembryonic development	Xing et al. 2008
	Haze_14427	chr5:30864351-30867761	TGdL_05 (B)	24.0-25.0 cM	Similar to RPK2: LRR receptor-like serine/threonine-protein kinase RPK2 (Arabidopsis thaliana OX3D3702)	Key regulator of anther development	Mizuno et al. 2007
	Haze_18449	chr7:13714444-13726805	TGdL_07	27.0-28.0 cM	Similar to HUA1: Zinc finger CCCH domain-containing protein 37 (Arabidopsis thaliana OX3D3702)	Binds AGAMOUS and promotes its processing	Cheng et al. 2003
	Haze_18603	chr7:18050699-18052517	TGdL_07	27.0-28.0 cM	Similar to EDA40: Probable E3 ubiquitin-protein ligase EDA40 (Arabidopsis thaliana OX3D3702)	Required for fusion of polar nuclei in the embryo sac	Pagnussat et al. 2005

Haze_18674	chr7:20000772-20016404	TGdL_07	27.0-28.0 cM	Similar to MED8: Mediator of RNA polymerase II transcription subunit 8 (Arabidopsis thaliana OX3D3702)	Component of the Mediator complex, regulates flowering time	Lalanne et al. 2004
Haze_11214	chr9:24175567-24181370	TGdL_09a	46.6.-50.1 cM	Similar to TCX2: Protein tesmin/TSO1-like CXC 2 (Arabidopsis thaliana OX3D3702)	Involved in both sexes' reproductive tissues	Sijiacic et al. 2011
Haze_11258	chr9:24770403-24775558	TGdL_09a	46.6.-50.1 cM	Similar to LFR: Armadillo repeat-containing protein LFR (Arabidopsis thaliana OX3D3702)	Required for the development of anthers	Wang et al. 2012
Haze_11325	chr9:25928569-25929273	TGdL_09a	46.6.-50.1 cM	Similar to SUP: Transcriptional regulator SUPERMAN (Arabidopsis thaliana OX3D3702)	Probable transcription factor that maintain the boundary between stamens and carpels	Hiratsu et al. 2002
Haze_11374	chr9:26557945-26563020	TGdL_09a	46.6.-50.1 cM	Similar to CRY2: Cryptochrome-2 (Arabidopsis thaliana OX3D3702)	Promotes the expression of FT and regulates negatively FLC	Endo et al. 2007
Haze_11453	chr9:27478530-27489055	TGdL_09a	46.6.-50.1 cM	Similar to CO3: Zinc finger protein CO3 (Oryza sativa subsp. japonica OX3D39947)	Control flowering time under short day conditions	Kim et al. 2008
Haze_11526	chr9:28361305-28376933	TGdL_09a	46.6.-50.1 cM	Similar to BLI: Protein BLISTER (Arabidopsis thaliana OX3D3702)	Required for normal flower development	Schatlowski et al. 2010
Haze_20664	chr10:19280716-19285541	TGdL_10a	16.6-18.1 cM	Similar to AP2-3: APETALA2-like protein 3 (Oryza sativa subsp. japonica OX3D39947)	Probable transcription factor that controls floral meristem establishment	Lee et al. 2007
Haze_20239	chr10:9829830-9836055	TGdL_10b	8.4-9.9 cM	Similar to CLO: 110 kDa U5 small nuclear ribonucleoprotein component CLO (Arabidopsis thaliana OX3D3702)	Splicing factor required for the female gametophyte development	Liu et al. 2009
Haze_20307	chr10:11538807-11544604	TGdL_10b	8.4-9.9 cM	Similar to RIE1: E3 ubiquitin protein ligase RIE1 (Arabidopsis thaliana OX3D3702)	Probable E3 ubiquitin-protein ligase required for embryo development	Xu and Li, 2003
Haze_13480	chr5:17056553-17065454	MB_05	53.0-54.0 cM	Similar to MADS1: Agamous-like MADS-box protein MADS1 (Vitis vinifera OX3D29760)	Probable transcription factor involved in flower development	Poupin et al. 2007
Haze_13578	chr5:19696883-19713247	MB_05	53.0-54.0 cM	Similar to JASON: Protein JASON (Arabidopsis thaliana OX3D3702)	Required for normal spindle orientation at male meiosis II and normal formation of tetrad of microspores	De Storme and Geelen, 2011
Haze_19677	chr10:2943733-2944491	MB_10	54.9-55.9 cM	Similar to OFP13: Transcription repressor OFP13 (Arabidopsis thaliana OX3D3702)	Transcriptional repressor that regulates late flowering	Wang et al. 2011

	Haze_19749	chr10:3637371-3643566	MB_10	54.9-55.9 cM	Similar to LIS: U4/U6 small nuclear ribonucleoprotein PRP4-like protein (Arabidopsis thaliana OX3D3702)	Involved in pre-mRNA splicing and it is required in female gametophyte for the egg cell and central cell fate determination	Voelz et al. 2012
Time of nut maturity (*tnm*)	Haze_03973	chr2:3333333-3335536	TGdL_02 (A)	14.5-18.0 cM	Similar to ACS3: 1-aminocyclopropane-1-carboxylate synthase 3 (Solanum lycopersicum OX3D4081)	Catalyzes the formation of a direct precursor of ethylene	Peng et al. 2005
	Haze_04056	chr2:3935750-3940413	TGdL_02 (A)	14.5-18.0 cM	Similar to GPA3: Protein GLUTELIN PRECURSOR ACCUMULATION 3 (Oryza sativa subsp. japonica OX3D39947)	Regulates trafficking of major storage proteins in seed endosperm during grain filling and maturation	Ren et al. 2014
	Haze_04470	chr2:8659452-8667683	TGdL_02 (B)	27.0-28.0 cM	Similar to EIN4: Protein EIN4 (Arabidopsis thaliana OX3D3702)	Redundant negative regulator of ethylene signaling	Hua et al. 1998
	Haze_25121	chr8:7061133-7061666	TGdL_08 (B)	38.0-40.0 cM	Similar to IND: Transcription factor IND (Arabidopsis thaliana OX3D3702)	Transcription regulator required for seed dispersal and involved in the differentiation of three cell types required for fruit dehiscence	Liljegren et al. 2004
	Haze_25134	chr8:7248982-7249212	TGdL_08 (B)	38.0-40.0 cM	Similar to IDL4: Protein IDA-LIKE 4 (Arabidopsis thaliana OX3D3702)	May be involved in floral abscission	Stenvick et al. 2008
	Haze_11440	chr9:27363951-27366788	MB_09	35.0-36.5 cM	Similar to ACS1: 1-aminocyclopropane-1-carboxylate synthase (Glycine max OX3D3847)	Catalyzes the formation of a direct precursor of ethylene	Peng at al.2005
	Haze_16946	chr11:27293393-27297388	MB_11	13.2-15.2 cM	Similar to AP2-2: APETALA2-like protein 2 (Oryza sativa subsp. indica OX3D39946)	Probable transcription factor involved in the control of inflorescence architecture and floral meristem establishment; also involved in grain growth	Dai et al. 2016
